# Economic and Disease Burden of Dengue Illness in India

**DOI:** 10.4269/ajtmh.14-0002

**Published:** 2014-12-03

**Authors:** Donald S. Shepard, Yara A. Halasa, Brij Kishore Tyagi, S. Vivek Adhish, Deoki Nandan, K. S. Karthiga, Vidya Chellaswamy, Mukul Gaba, Narendra K. Arora, the INCLEN Study Group

**Affiliations:** Brandeis University, Waltham, Massachusetts; Centre for Research in Medical Entomology, Madurai, India; National Institute of Health and Family Welfare, New Delhi, India; INCLEN Trust International, New Delhi, India

## Abstract

Between 2006 and 2012 India reported an annual average of 20,474 dengue cases. Although dengue has been notifiable since 1996, regional comparisons suggest that reported numbers substantially underrepresent the full impact of the disease. Adjustment for underreporting from a case study in Madurai district and an expert Delphi panel yielded an annual average of 5,778,406 clinically diagnosed dengue cases between 2006 and 2012, or 282 times the reported number per year. The total direct annual medical cost was US$548 million. Ambulatory settings treated 67% of cases representing 18% of costs, whereas 33% of cases were hospitalized, comprising 82% of costs. Eighty percent of expenditures went to private facilities. Including non-medical and indirect costs based on other dengue-endemic countries raises the economic cost to $1.11 billion, or $0.88 per capita. The economic and disease burden of dengue in India is substantially more than captured by officially reported cases, and increased control measures merit serious consideration.

## Introduction

Dengue, an arboviral infection transmitted by *Aedes aegypti* and *Aedes albopictus* mosquitoes, is emerging as the most important mosquito-borne viral disease. It is a serious global public health problem, with 2.5 billion people at risk and an annual range of 50 to 390 million infections, which include dengue fever, dengue hemorrhagic fever (DHF), and dengue shock syndrome (DSS).^1^–^5^ Climate change, the expansion of dengue vectors to new geographic regions, increasing human movement across borders, global trade, and urban migration collectively have changed the scope and scale of dengue fever from a national to a global concern.^2^,^4^,^6^–^9^ The increasing number of dengue cases imported from endemic countries and autochthonous transmission of dengue in non-endemic areas such as the United States (Hawaii, Florida, and Texas),^10^,^11^ France,^12^ Croatia,^13^ and Angola^14^ highlight the importance of global collaboration to manage dengue epidemic.

India's notable 2.1% share of global international travel in 2012,^15^ its increasing role in the global economy,^16^ and its growing public health problem with dengue^17^,^18^ call for a closer look at the dengue challenge. Although dengue has been notifiable in India since 1996, the disease's impact has been underestimated because of insufficient information on incidence and cost of dengue illness.^19^ Between 2006 and 2012 the National Vector Borne Diseases Control Program reported an annual average (± standeard deviation [SD]) of 20,474 (±13,760) dengue cases and 132 (±57) deaths caused by dengue.^20^ Regional comparisons suggest that these official numbers reflect only a small fraction of the full impact of the disease.^18^,^21^,^22^ Estimates of the average annual number of cases vary widely from the 20,474 officially reported cases to an annual 33 million apparent cases.^2^ India's selective surveillance system reports cases from 347 Sentinel Surveillance Hospitals with full-fledged laboratories and 14 Apex Referral Laboratories. Reported dengue cases have increased dramatically over the study period. Annual cases doubled repeatedly from 10,137 for 2006–2008 to 20,896 for 2009–2011 to 47,029 in 2012. State-by-state comparisons across these time periods showed that 74% of the 68 possible comparisons reflected increases compared with 21% with decreases and 6% with no change. This pattern likely reflects both real increases in dengue and improved reporting. Nevertheless, reported data do not adjust for cases treated outside of Sentinel Surveillance Hospitals or for the inability of typical dengue tests (e.g., immunoglobulin M [IgM]) to detect recent dengue infections. Understanding the economic and disease burden of dengue in India is essential to assist policy makers and public health managers to prepare for and control outbreaks, and encourage international collaboration to develop and evaluate prevention, control and management measures, and technologies to control further epidemics.^18^,^23^

The first isolation of dengue in India occurred in Calcutta in 1945–46. India's first dengue fever epidemic was reported in 1963–64, when dengue gradually spread from the country's southern regions to its northern states and progressively to the whole country by 1968.^6^ Since then, India has experienced extensive dengue epidemics each followed by endemic/hyper-endemic years, with a shift in dengue epidemiology in 1996 introducing the first major epidemic of DHF/DSS.^6^ Trends in recent decades indicate that larger and more frequent dengue outbreaks are occurring, with geographic expansion to new states, and spread of dengue to peri-urban and rural areas, in addition to increased case severity and deaths, and progression to hyper-endemicity.^20^,^23^,^24^ Cost of illness information is needed to quantify the present problem, to evaluate the benefit and effectiveness of various prevention and control technologies, and to identify appropriate combinations of strategies to control dengue.

Although a few studies addressed the economic burden on dengue in India,^25^,^26^ they lacked systematic empirical data on treatment setting and adjustment factors to correct for underreporting. Our literature search did not identify any published or public unpublished reports on seroprevalence studies in India. To address these gaps, we conducted a national retrospective study based on a retrospective hospital-based study, a prospective pilot study, a district case study, and existing surveillance studies to estimate the impact of dengue in India. The study framework and methodology were published previously.^19^ Our three-part study estimates the 1) direct medical cost of a dengue episode based on a 10-site retrospective study with unit cost analyses plus a pilot prospective study, 2) numbers of dengue cases based on a district-level case study and a Delphi panel, and 3) aggregate cost of dengue by setting and sector combining all sources.

## Methods

### Part 1: Direct medical cost of a dengue episode.

To estimate the direct medical cost of a dengue episode, we conducted a retrospective study of clinically diagnosed hospitalized dengue cases in 10 leading medical colleges in India and a pilot prospective study at one of these sites. The cost of a hospitalized day and cost of an ambulatory visit were estimated using the macro-costing approach at all 10 sites. The pilot survey interviewed a sample of patients who received care at the study hospital in Mumbai and its associated urban health center.

#### Retrospective study.

To capture India's geographic and socio-cultural diversity for the retrospective study, we divided the country into five regions. In each region, we selected two medical colleges located in separate states, as shown in Table 1. The medical colleges were selected based on the interest of a senior medical professor to lead the study, the professor's and hospital's willingness and ability to commit the necessary personnel time, and prior collaboration history. These hospitals discharged 4,125 patients with a clinical diagnosis of dengue during the study years (2006 through 2011). Our sample of patients for analysis consisted of all dengue patients from that hospital in a given year if 50 or fewer were discharged, or a random sample of the dengue discharges from that hospital in that year if there were more than 50. This process identified 1,665 dengue discharges for study. We developed a data abstraction instrument to record needed data from these selected discharges, piloted at Lady Harding Medical College in New Delhi, and modified according to recommendations made by the sites' principal investigators. Items included the number of ambulatory visits the patient received before hospitalization, source of admission, length of stay, reported signs and symptoms, and type and number of imaging and laboratory tests conducted.

Under the supervision of the site principal investigator, a researcher with a community medicine background requested the selected medical records from the hospital's medical record department, completed the data extraction instrument for each record found, entered these data into an Excel spreadsheet, and sent both hard copies and the Excel files to Center for Research in Medical Entomology (CRME) for cleaning, consistency checks, and analysis. A researcher at CRME entered the data from all cases into EpiData (version 3.0), validated the data against the Excel file developed by the hospital research assistant, and exchanged requests for clarification and information with the study hospitals as needed. In an attempt to identify possible underdiagnosis of dengue at these hospitals, we also selected and abstracted records from patients with fever of unknown origin and other specified febrile illnesses.^19^

#### Pilot interview study.

We complemented the retrospective study with a pilot survey interviewing a sample of patients with febrile illness who sought care at an urban health center and a public medical college hospital in Mumbai between November 2012 and March 2013. This pilot allowed us to fill gaps in the data collected from the medical records, specifically the number and type of ambulatory visits a patient sought during a hospitalized dengue episode, and illustrated the number and type of ambulatory visits sought by dengue patients treated only in an ambulatory setting.

To cover the full course of a dengue episode we conducted two interviews: the first was during the illness episode and the second after recovery. The questionnaire was translated from English into Hindi and Marathi. We conducted the first interview on 151 patients (120 hospitalized and 31 ambulatory cases). To determine the representativeness of respondents to the second interview, we tested for differences in demographics between those who completed the first interview only compared with those who finished both interviews.

#### Cost of a dengue episode.

We collected information related to operational budget, bed capacity, occupancy, and aggregate ambulatory visits from 10 medical colleges for the fiscal years 2010–2011 and 2011–2012. The operational budget for each hospital was classified into nine main categories, i.e., hospital staff, doctors, materials, drugs, equipment, vehicles, other inputs (utilities and maintenance), inputs provided by other agencies (such as staff housing), and user fees used for patient care. We used the results from Chatterjee^27^ to estimate depreciation of the building. We estimated missing items as the product of that hospital's volume (bed day equivalents) times the median cost per bed day equivalent for the missing item in remaining hospitals by sector (private, public). We used the macro costing method to estimate the cost per bed day and cost of an ambulatory visit.^28^ After adjusting for inflation, the average cost per bed day equivalent and cost per ambulatory visit for the two fiscal years were used to estimate the cost of dengue illness for the year 2012.

### Part 2: Number of dengue cases in India.

To estimate the disease burden of dengue in India, we conducted an empirical case study in the district of Madurai in the state of Tamil Nadu to derive an adjustment factor to calculate the true number of clinically diagnosed dengue patients in that district. Results from this case study were complemented by expert opinion and results from the retrospective study we conducted at the 10 medical colleges. To extrapolate the adjustment factor to the national level we used surveillance data obtained at the state and national level.

#### Delphi panel and experts' opinion.

Fifty national and international experts participated in a 1-day workshop in February 2013 in New Delhi, India, to share and discuss current knowledge on dengue in India. Representing government, academic and private perspectives, these experts came from participating institutions (e.g., the Indian Council for Medical Research), national and state health agencies (e.g., the Integrated Disease Surveillance Project), and private organizations (e.g., laboratories). Nine of these experts constituted an anonymous Delphi panel. These experts wrote their best estimate of the percentage of dengue cases treated in an ambulatory setting, were told the summary statistics (median, minimum, and maximum), and discussed their estimates, and repeated the process in a second round. We then derived the best estimate and confidence interval (CI) from their final summary statistics using a triangular distribution generated through 1,000 Monte Carlo iterations.

#### Adjustment factor: Madurai district case study.

We developed a descriptive inventory of all healthcare facilities treating dengue patients in Madurai district. The inventory classified healthcare units by sector into private and public based on their statutory ownership, and setting into ambulatory facilities and hospitals. It stratified private hospitals according to their bed capacity into three groups: small hospitals (capacity of 1 to 50 beds), moderate hospitals (capacity of 51 to 100 beds), and large hospitals (capacity of more than 100 beds). We obtained numbers of hospitalized clinically diagnosed dengue cases tested with IgG, IgM, or NS1 for the years 2009 through 2011 from Madurai Medical College public laboratory for public hospitals, and from a stratified sample of 12 of the 250 private hospitals operating in Madurai district during the study period. By using this 3-year period, we controlled for fluctuations in dengue awareness and reporting (higher in epidemic years and lower in other years).

Interviews with dengue experts and healthcare providers indicated that some clinically diagnosed dengue cases were not tested. We adjusted for this limitation by using four sources. First, from the retrospective study we calculated the share of hospitalized patients with a dengue discharge tested for dengue using IgG, IgM, or NS1 from 2009 through 2011 (71% of such patients in public tertiary hospitals and 90% of such patients in private tertiary hospitals). Second, using expert opinion, we estimated that 25% of hospitalized dengue cases in other public hospitals and 50% of hospitalized dengue cases in other private hospitals were tested for dengue. Third, using the number of beds in Madurai by type of hospital, we extrapolated the total number of hospitalized dengue cases in Madurai. Finally, using the average estimate determined by experts participating in the Delphi panel of the proportion of dengue patients treated at an ambulatory setting (67%, CI: 47–87%), we determined the number of ambulatory cases in Madurai. We compared projected dengue cases from the Madurai case study with the officially reported numbers from the district surveillance unit to the state level. We then adjusted the state-level estimate for the share of cases tallied at the national level.^29^

#### Adjustment factor: national and state surveillance data.

We requested national and state dengue surveillance data for all 35 states and union territories in India for the years 2006 through 2012.^20^ We obtained complete national data, state data for 18 states, and compared these state data against the corresponding national information. Combining our results with the adjustment factor derived from the Madurai case study and expert opinion, we estimated both the reporting rate and the adjustment factor at the state and national levels for India.

### Part 3. Aggregate direct medical cost of dengue in India.

To estimate the aggregate direct medical cost of the dengue in India, we first derived the average annual projected number of dengue cases for the years 2006–2012 based on the Madurai study and expert opinion. From macro-costing we estimated the cost of a dengue case by setting (ambulatory versus hospitalization) and sector (public versus private).

#### Computational procedures.

A hospitalized case uses ambulatory services in addition to the hospital stay. To compute the cost of a dengue hospitalization for each sector, we multiplied the average length of stay for dengue patients obtained from the retrospective study by the cost per bed day equivalent obtained from the macro costing. We calculated the cost of ambulatory services based on number and type of ambulatory visits obtained from the pilot study conducted in Mumbai. We then summed the cost of the hospital stay and associated ambulatory visits. To compute the cost of cases receiving only ambulatory care, we used the number and types of visits from the Mumbai pilot survey. To estimate the aggregate cost, we computed the weighted average cost of the annual projected dengue cases by sector and setting.

#### Sensitivity analysis.

To calculate CIs we used the probabilistic sensitivity analysis with triangular distributions for 1) the share of cases treated in the hospital sector, 2) the adjustment factor, 3) the share treated in the private sector, 4) cost per day in private hospitals, 5) cost per day in public hospitals, 6) length of stay in private hospitals, and 7) length of stay in public hospitals. Available data for each parameter generated the minimum, maximum and best estimate. For each sensitivity analysis, we performed 1,000 Monte Carlo iterations with independent drawings for each parameter. We presented results as mean values and 95% CIs in a tornado diagram.

### Ethics statement.

For the retrospective study, data were collected entirely retrospectively from medical records, with no contact with patients. Analysts had access only to the patient's study number, with no access to the patient's medical record, name, or phone number. For the pilot study, the study was described to participants and a written consent was signed before the first interview. Participants who completed the second interview were offered an appreciation gift equivalent to US$5.00 for their time. The study methods and tools were approved by the Institutional Ethical Committee of CRME, the INCLEN Independent Ethics Committee, the International Division of the Ministry of Science and Technology, and the Institutional Review Board at Brandeis University.

## Results

### Part 1: Direct medical cost of a dengue episode.

#### Services received per dengue episode.

From the 1,665 sampled dengue discharges, we obtained and reviewed 1,541 medical records; these form the basis for this analysis. The remaining medical records were missing or could not be retrieved as a result of the storage arrangement of older medical records. The age distribution of the final sample was 20.2% < 5 years of age, 35.4% between the ages of 5 and 15, and 44.4% adults (16 + years). Most of the cases came from urban settings (49.0% from established urban areas and 9.0% slums or resettlements), with the remainder from rural areas (41.6%) and unknown locations (0.4%). This pattern indicated that dengue might be spreading to rural areas as well. The inpatient case-fatality rate was 4.6%.

On average (±SD), patients were admitted 6.51 (±5.24) days after the onset of illness. The duration of documented illness (start of illness through discharge) was, on average, 12.16 (±10.00) days. The average length of stay was 5.65 days (±4.12). The length of stay in private hospitals (6.47 ± 1.18) days was about 1 day longer than that in public hospitals (5.37 ± 0.76 days). Forty-eight percent of these patients were admitted for reasons other than dengue; of these cases 51.7% were admitted as either acute febrile illness (32.3%) or fever of unknown origin (19.4%). The diagnosis of dengue cases at discharge classified 83.9% of cases as dengue fever, 9.5% as DHF, 5.9% as DSS, and 0.7% as co-morbidity of dengue and another infection, such as typhoid or malaria. Between 2006 and 2011, 87.7% of patients with a clinical dengue diagnosis treated in private medical colleges were tested for dengue, compared with 72.8% at public medical colleges. Retrospective analysis of patients with fever of unknown origin or other specified febrile illnesses did not identify probable dengue cases, as the medical records did not contain the needed details,^19^ such as dengue tests or chronology of fever and other signs and symptoms.

#### Pilot interview study.

Of the 151 patients who consented to participate in the pilot study, 50 patients completed both interviews, giving an overall response rate of 33%. The remaining patients were either lost for follow-up (*N* = 27, 18%) or their blood sample was insufficient to perform the additional dengue test (*N* = 75, 49%). The cooperation rate was 100%, as all patients with a valid blood sample participated in a second interview. The respondents comprised both hospitalized (*N* = 31) and ambulatory (*N* = 19) patients. On average (±SD), the first interview took place 6 (±5) days after the onset of the illness episode. The second interview was conducted on average 58 (±31) days after the first interview. Only a few (*N* = 6, 12%) second interviews were conducted in person; most were conducted by telephone (*N* = 44, 88%). Our tests for representativeness of the 50 second-interview participants compared with the 151 first-interview respondents found no significant difference in age, education, being a student, working for pay, or symptoms (including myalgia, nausea, retro-orbital pain, chills, cold, cough, generalized weakness, abdominal pain, vomiting, headache, and body-ache).

All patients were fully recovered from their illness episode by the date of the second interview. On average (±SD), the fever lasted for 5.8 ± 3.7 days from the onset of the illness. For ambulatory patients the fever ended in 7.4 ± 3.8 days, compared with 4.8 ± 3.3 days for hospitalized cases. This difference was statistically significant (t[48] = 2.56, *P* = 0.014). The overall duration of illness was 8.7 ± 13.0 days for ambulatory cases and 11.4 ± 20.6 days for hospitalized cases. The difference in the illness duration between ambulatory and hospitalized cases was not statistically significant (t[48] = 0.57, *P* = 0.57).

On average, ambulatory patients had 2.53 ± 0.90 visits with health providers, comprised of 1.26 ± 0.56 in a hospital outpatient department, 0.95 ± 0.91 in a private clinic, 0.16 ± 0.50 in a pharmacy, 0.11 ± 0.32 in a community health center, and 0.05 ± 0.23 in an emergency room. On average hospitalized cases had 1.62 ± 0.80 ambulatory visits, consisting of 0.61 ± 0.62 visits to a hospital outpatient department, 0.52 ± 0.77 to an emergency room, 0.23 ± 0.43 to a pharmacy, 0.23 ± 0.43 to a private clinic, and 0.03 ± 0.18 to a primary health care center. The difference in number of ambulatory visits between hospitalized and ambulatory cases was highly statistically significant (t[48] = 3.66, *P* < 0.001).

#### Cost of dengue episode.

We obtained cost data from all 10 medical college hospitals. The average (±SD) cost per bed day was $35.66 ± 10.62 in the three private-for-profit medical colleges and $32.11 ± 12.31 in the seven publicly owned and managed medical colleges. The cost of a hospitalized episode in the public sector averaged $197.03, consisting of $181.40 for the hospital stay, $11.61 for 1.13 hospital outpatient visits, and $4.03 for 0.49 other ambulatory visits. The mean cost of a hospitalized episode in the private sector was $248.11, consisting of $230.74 for the inpatient stay and $17.37 for ambulatory visits. The average cost of an ambulatory episode was $26.09 if treated in the private sector and $23.49 if treated in the public sector.

### Part 2: Number of dengue cases in India.

#### Delphi panel and experts' opinion.

On average (±SD) the panel estimated that 67% (±17%) of all dengue patients were treated only in an ambulatory setting with a range of 40–90%, and a CI of 47–87%.

#### Adjustment factor: Madurai case study.

From 2009 through 2011, an annual average of 6,334 clinically diagnosed hospitalized dengue cases from Madurai were referred to a microbiology department to be tested with IgG, IgM, or NS1 for dengue. The public sector was the source of 24.23% and the private sector was the source of 75.77% of these cases. For the same period, the average number of reported dengue cases at the district level was 134 cases, and 126 cases at the state level. After applying the adjustment factors, the average annual number of all clinically diagnosed hospitalized dengue cases was estimated for Madurai district at 11,975 hospitalized cases. Of these patients, 24% were treated in the public sector and the remaining (76%) in the private sector. From the results of the Delphi panel, we estimated that hospitalizations represented only 33% (CI: 13–53%) of all symptomatic dengue cases in the district. Our calculation yielded 36,287 clinically diagnosed dengue cases (CI: 22,589–92,215) of which 24,312 (CI: 10,619–80,240) were ambulatory cases. Thus, state reporting in Tamil Nadu captured only 0.35% (range: 0.14–0.56%) of these clinically diagnosed dengue cases, with an adjustment factor of 288 (range: 176–717), as shown in Table 2.

#### Adjustment factor: national and state surveillance data.

The annual average number of reported dengue cases at the national level in India for the years 2006 through 2012 was 20,474. A comparison of the available data from 18 states showed that the state reports averaged 2% below the corresponding national reports. The differences between these levels are probably caused by the restrictions imposed on the criteria for dengue reporting (e.g., some states excluded cases confirmed by rapid test, although the national level included them) and results of imported and exported cases (patients treated outside their state of residence).

**Figure 1.**
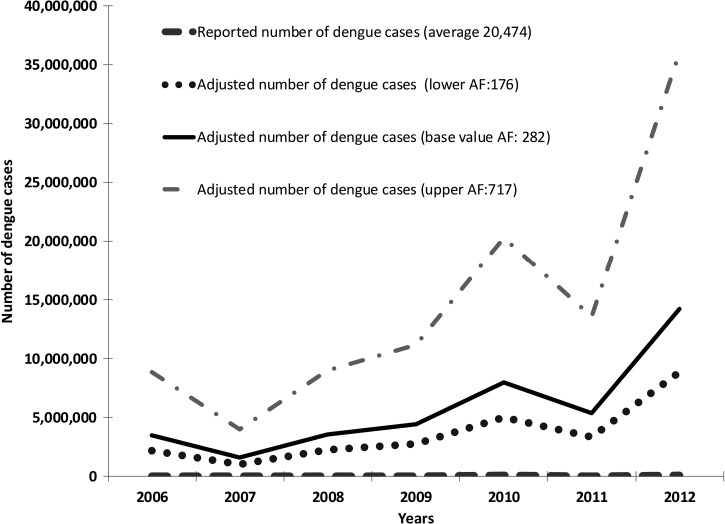
Reported and adjusted number of clinically diagnosed dengue cases, 2006–2012. AF denotes adjustment factor.

Combining the results from the Madurai case study and the expansion factor at the national level with the ratio (reporting at state level/reporting at national level) of 0.98 yielded a national expansion factor of 282 (CI: 176–717) for India. Therefore, the projected annual average of dengue cases in India is 5,778,406 (CI: 3,597,174–14,684,499), in stark contrast to the reported number 20,474, as shown in Table 2 and Figure 1.

### Part 3. Aggregate direct medical cost of dengue in India.

Table 3 presents the aggregate direct medical cost of dengue in India. The total annual aggregate cost was US$547 million (range: $341 million–$1,390 million). Of this cost, 81.9% was for hospitalized cases and 18.1% for ambulatory cases. The share of the private sector was 75.8% compared with 24.2% in the public sector. The excess cost of a case in the private sector compared with the public sector was 26% if hospitalized and 11% if ambulatory.

**Figure 2.**
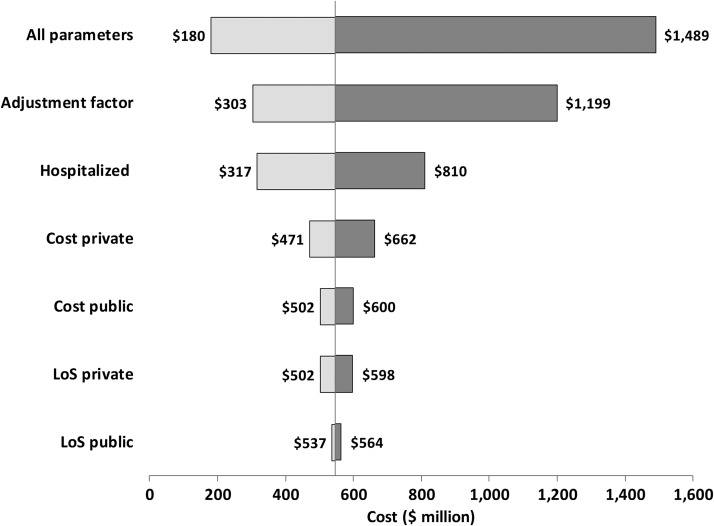
Sensitivity analyses of aggregate medical cost of dengue illness and variation according to key costing parameters, millions of 2012 US$. LoS denotes length of stay. The central value is $548 million.

As shown in Figure 2, three parameters were associated with substantial variation on the aggregate direct medical cost of dengue. The adjustment factor can reduce the cost by 45% or increase the cost by 119% compared with the mean value. The variation in percentage of dengue cases treated in the hospital setting could reduce the estimate by 42% or increase it by 48% compared with the base value. Variation in the mix of cases by sector could reduce costs by 14% of the base value if all cases were treated in the public sector, or increase costs by 21% if all cases were treated in the private sector.

In summary, the total direct medical cost of dengue in 2012 was $548 million or $0.43/capita. Payment for treatment in the private sector for both hospitalized and ambulatory services represented 80% of this total cost or $0.35 per capita. The overall average medical cost of a dengue case was $235.20 if hospitalized, $25.46 if entirely ambulatory, and $94.24 overall. Our results suggest that 80% of this cost is paid for through private sources, mainly households, and 20% is covered by public sources.

## Discussion

To our knowledge, this is the first study to estimate the disease burden and direct medical cost of dengue in India using empirical data. Between 2006 and 2012, India reported an annual average of 20,474 dengue cases. Our results suggest that after correcting for underreporting, India had nearly 6 million clinically diagnosed dengue cases for the same period; so for each reported dengue case at the national level, 282 clinically diagnosed dengue cases actually occurred and obtained medical care. According to our study, the National Vector Borne Diseases Control Program captures only 0.35% of the annual number of clinically diagnosed dengue cases in India. The dengue reporting rates derived from our study are comparable to the low reporting rates from India of deaths associated with rabies (1.23%),^30^ and malaria (0.38–0.75%),^31^and cases of chikungunya (3.7%)^32^ and malaria (13.4%).^33^

Although an estimate from a previous study based primarily on geographical factors suggests an expansion factor for “apparent infections” of 1,166,^2^ our district level study found a factor of 282 for “clinical cases.” Part of the 4-fold difference may lie in a reporting bias in data used for the former estimate. Years and locations with dengue outbreaks or suspected concentrations of dengue may be more apparent, easier to study, and more likely to be published than data from similar settings and years without outbreaks. Furthermore, apparent infections for which no medical care is sought would be included in the expansion factor of 1,166 but not in the factor of 282 derived here. Additionally, the sites where dengue sero-prevalence data existed for the geographically based study were likely ones where epidemiological investigators suspected that dengue was concentrated.

Madurai district appeared to be representative of the rest of the state of Tamil Nadu, with 125 reported annual dengue cases in 2006–12 compared with an average of 126 in all other districts (Tamil Nadu Department of Health, 2013, unpublished data). Among India's 35 states and territories, Tamil Nadu reported the highest percentage of dengue cases (14%) in 2006–12 as a result of the state's well-established surveillance system, the endemicity of dengue, and well-informed health workers.^20^ As dengue reporting is likely better in Tamil Nadu than elsewhere in India, the adjustment factor of 282 may even be low. Although we were able to collect only the number of hospitalized dengue cases tested for dengue in Madurai, the complementary data sources for this project allowed us to adjust for this limitation.

Because a dengue case averages 2 weeks, its medical cost per day of illness is about $6.77 ($94.85/14 days). By comparison, a case of tuberculosis in India averages 72 days and costs (adjusted to 2011 prices) $241 to $281 or $3.39 to $3.96 per day.^34^,^35^ Thus, per day of illness, dengue costs twice as much as one of tuberculosis.

The direct medical cost of illness is only part of its overall economic cost. The overall cost also includes direct non-medical costs (e.g., travel) and indirect costs (the value of time lost from morbidity and premature death). The latter components tend to be borne especially by households.^36^ In a study of dengue costs across eight low- and middle-income countries, direct medical costs averaged 66% of overall economic costs for hospitalized episodes and 23% of overall costs for ambulatory episodes.^37^ Assuming India had similar percentages; direct medical costs would represent 49% of overall illness costs in India. Therefore, overall annual economic costs of dengue illness in India would be about $1.11 billion or $0.88/capita.

Our study has several limitations. 1) Our empirical estimate of the expansion factor is based on only one district: Madurai. 2) Within our Madurai case study, data were based only on tested dengue cases, as facilities were not authorized to release reports based on untested dengue cases. Indeed, some anecdotes suggested that even if the cases were clinically diagnosed as dengue, health providers were reportedly advised to report them as pyrexia of unknown origin (private discussion with health providers and experts, 2013). 3) Although our inventory of health providers in Madurai suggested that Madurai district had 154 private clinics in 2011, we were not able to collect the number of dengue patients retrospectively from these clinics in that year because of a lack of documentation. However, we did collect the number of dengue patients from a sample of these clinics for the year 2012, and outpatient departments from private hospitals, public health centers, and maternal centers. 4) Our data used for costing (length of hospital stay and unit costs) were based on only 10 medical college hospitals in India. However, these hospitals were selected from 10 separate states in India, spanning all geographic regions of the country and both private and public facilities. 5) Although we succeeded in including the private sector in our study, where literature suggests that 80% of all services are provided,^18^ data from ambulatory settings was scarce. 6) Our data on ambulatory services received were based only on the pilot study linked to just one hospital. 7) Our data from the hospitalized cohort were based on clinical diagnoses. As the majority of patients were laboratory confirmed, specificity is likely to be high, but some false negatives are likely because IgM remains negative for the first few days of fever and dengue can be difficult to distinguish from other febrile illnesses from clinical signs alone.

We endeavored to address these limitations through examining records for patients with other febrile illnesses, performing sensitivity analyses (e.g., a range of unit costs in both the public and private sectors) and using complementary data sources. Consistency across sources generated confidence in our findings. For example, the average number of ambulatory visits accrued by the hospitalized dengue cases in our Mumbai pilot study (1.62 visits) was close to the number of ambulatory visits extracted from the medical records of hospitalized dengue patients in 10 medical colleges (1.55 visits). However, both of these averages are lower than numbers of visits from dengue patients in other low- and middle-income countries.^37^ Further studies, including prospective designs, are needed to refine the economic burden of dengue treated in an ambulatory or informal setting or misdiagnosed, and the indirect and social costs of dengue.

Our results indicate that the economic and disease burdens of dengue in India are hundreds of times greater than estimates based entirely on official reporting. The majority of costs are incurred in the private sector and are paid mostly by households. With India's increasing role in international travel and the global economy, the need for additional dengue control and prevention strategies when available, such as vaccine and innovative vector control measures, becomes increasingly strong.

## Figures and Tables

**Table 1:** States and medical colleges in retrospective study

Region	States in region	Selected states, medical colleges, and cities
North	Delhi, Rajasthan, Jammu and Kashmir, Himachal Pradesh, Punjab, Haryana, Uttarakhand and Chandigarh	NCT of Delhi (Lady Harding Medical College, Delhi), Rajasthan (NIMS Medical College and Hospital, Jaipur)
South	Tamil Nadu, Karnataka, Kerala, Andhra Pradesh, Puducherry, Lakshadweep, Andaman and Nicobar	Karnataka (Kasturba Medical College, Manipal), Tamil Nadu (Madras Medical College, Chennai)
East	Sikkim, Assam, Tripura, Manipur, Nagaland, Arunachal Pradesh, Mizoram, Meghalaya, Bihar, Jharkhand, West-Bengal and Orissa	Manipur (Regional Institute Medical Sciences, Imphal), Orissa (Kalinga Institute of Medical Sciences, Odisha)
West	Dadar and Nagar Haveli, Daman and Diu, Goa, Maharashtra and Gujarat	Gujarat (M.P. Shah Medical College, Jamnagar), Maharashtra (L.T.M. Medical College and General Hospital, Mumbai)
Central	Uttar Pradesh, Chhattisgarh, and Madhya Pradesh	Uttar Pradesh (Integral Institute of Medical Sciences and Research, Lucknow), Madhya Pradesh (G.R. Medical College, Gwalior)

**Table 2:** Adjusted annual average number of dengue cases, 2006–2012*

Parameters	Estimates
Madurai District (average 2009–2011)	
Hospitalized dengue cases	11,975
Tested with NS1, IgG, IgM	6,334
Untested with NS1, IgG, IgM	5,641
Ambulatory dengue cases	24,312
	[10,615–80,138]
Clinically diagnosed dengue cases (Madurai district)	36,287
	[22,589–92,215]
IDSP reported cases	134
Adjustment factor at IDSP level	271
	[165–672]
State reported cases	126
Adjustment factor at State level	288
	[179–732]
Adjustment factor at the national level	0.98
Overall adjustment factor for India	282
	[176–717]
Number of reported dengue cases at the national level (average 2006–2012)	20,474
Adjusted number of dengue cases at the national level (average 2006–2012)	**5,778,406**
	**[3,597,174–14,684,499]**

* Notation: IDSP denotes Infectious Disease Surveillance Program; [–] denotes ranges.

**Table 3:** Annual direct medical cost of dengue in India by sector and setting, 2012 USD*

	Public	Private	Total
Cost of a hospitalized case			
Cost per night	$32.11	$35.66	$34.80
Average length of stay	5.65	6.47	6.27
Cost per inpatient stay	$181.40	$230.74	$218.25
Cost of ambulatory visits	$15.64	$17.37	$16.95
Total cost per hospitalized episode	$197.03	$248.11	$235.20
Projected annual number of hospitalized cases	462,029	1,444,845	1,906,874
	[287,622–1,174,140]	[899,445–3,671,744]	[1,187,067–4,845,885]
Aggregate cost of hospitalized cases	**$91,035,000**	**$358,478,000**	**$449,513,000**
	**[$56,671,000–231,344,000]**	**[$223,160,000–910,990,000]**	**[$279,831,000–1,142,334,000]**
Cost of an ambulatory case			
Total ambulatory cost per episode	$23.49	$26.09	$25.46
Projected annual number of ambulatory cases	938,058	2,933,474	3,871,532
	[583,960–2,383,861]	[1,826,146–7,454,753]	[2,410,106–9,838,614]
Aggregate cost of ambulatory cases	**$22,031,000**	**$76,529,000**	**$98,560,000**
	**[$13,715,000–55,987,000]**	**[$47,641,000–194,480,000]**	**[$61,356,000–250,467,000]**
Total aggregate cost by sector	**$113,066,000**	**$435,007,000**	**$548,073,000**
	**[$70,386,000–287,331,000]**	**[$270,801,000–1,105,470,000]**	**[$341,187,000–1,392,801,000]**

* [–] denotes 95% confidence intervals.
